# Metagenomic Characterization of Poultry Cloacal and Oropharyngeal Swabs in Kenya Reveals Bacterial Pathogens and Their Antimicrobial Resistance Genes

**DOI:** 10.1155/2024/8054338

**Published:** 2024-02-12

**Authors:** Philip M. Panyako, Sheila C. Ommeh, Stephen N. Kuria, Jacqueline K. Lichoti, Johns Musina, Venugopal Nair, Vish Nene, Muhammad Munir, Samuel O. Oyola

**Affiliations:** ^1^Institute for Biotechnology Research, Jomo Kenyatta University of Agriculture and Technology, Nairobi, Kenya; ^2^Directorate of Veterinary Services, State Department of Livestock, Ministry of Agriculture, Livestock and Fisheries, Nairobi, Kenya; ^3^Department of Zoology, National Museums of Kenya, Nairobi, Kenya; ^4^Pirbright Institute, Woking, Surrey, UK; ^5^International Livestock Research Institute (ILRI), Nairobi, Kenya; ^6^Department: Biomedical and Life Sciences, Lancaster University, Bailrigg, UK

## Abstract

Poultry enteric bacterial diseases are of significant economic importance because they are responsible for production losses due to weight loss, increased morbidity and mortality, and increased cost of production arising from poor feed conversion and treatment. This cross-sectional purposive study characterized enteric bacterial pathogens in poultry from selected agroclimatic regions in Kenya and investigated their antimicrobial resistance gene profiles. Cloacal (*n* = 563) and oropharyngeal (*n* = 394) swabs were collected and pooled into 16 and 14 samples, respectively, to characterize bacterial pathogens and their antimicrobial resistance gene profiles. We report that *Proteobacteria*, *Chlamydiae*, and *Firmicutes* are the most dominant phyla present in both cloacal and oropharyngeal swabs of the six poultry species studied, indicating the colonization of the poultry gut by many pathogenic bacteria. Using KEGG and COG databases, some pathways related to metabolism, genetic information, and cellular processing were detected. We also report the abundance of antimicrobial resistance genes that confer resistance to *β*-lactamases, aminoglycosides, and tetracycline in most of the poultry analyzed, raising concern about the dangers associated with continuous and inappropriate use of these antibiotics in poultry production. The antimicrobial resistance gene data generated in this study provides a valuable indicator of the use of antimicrobials in poultry in Kenya. The information generated is essential for managing bacterial diseases, especially in backyard poultry raised under scavenging conditions.

## 1. Introduction

Poultry farming is practiced in many parts of the world because of its economic importance. Poultry meat and egg production is a source of livelihood for farmers and a major protein source for consumers. The major reasons for the popularity of poultry farming are the minimal religious and cultural restraints on their consumption, in addition to their relatively low costs of production [1]. Currently, poultry meat is the most widely consumed meat type, accounting for 35% of the meat consumed globally [2].

The most common poultry raised in Kenya include chickens, ducks, guinea fowls, quails, geese, turkeys, pigeons, and ostriches. Three methods are used for rearing poultry: the free-range system, the semi-intensive system, and the intensive system. In Kenya and other developing countries, poultry is mainly raised under free-range (scavenging) systems in rural settings; hence, it is commonly referred to as village poultry [3]. The free-range or backyard system is popular in rural areas because it is less capital-intensive and applies little to no biosecurity measures [4]. In addition, indigenous African poultry are known to be more tolerant to diseases and harsh environmental conditions than commercial chickens, comprising heterogeneous populations [5].

The demand for poultry products has pushed many farmers to intensify poultry production over the last century, resulting in rapid growth in the industry. The current poultry biomass, for instance, accounts for approximately 70% of the total biomass of birds worldwide [6, 7]. Poultry flocks are often kept in high-density populations that are genetically homogeneous. This potentially makes them susceptible to outbreaks of infectious diseases, leading to significant economic losses and food insecurity [7]. The extensive utilization of the same antimicrobial classes in humans as well as veterinary medicine (such as treatment and growth promotion in poultry) is also contributing to antimicrobial resistance (AMR) selection, which is a major public health concern [8]. There is therefore a need to characterize bacterial pathogens in backyard poultry and also profile the antimicrobial resistance genes (ARGs) in these poultry species.

Several approaches have been employed for studying poultry gut microbiota, with the earliest being the culture-based methods [9, 10]. Unfortunately, these methods were prone to bias and inaccuracy as most microorganisms were not cultivatable because of unknown growth requirements [1]. Several polymerase chain reaction (PCR)-based techniques have also been exploited for evaluating microbial profiles and detecting antimicrobial resistance genes in poultry, such as the Sanger sequencing technology. Although these methods improved the sensitivity and speed of detection of microorganisms and their antimicrobial resistance genes, they were still unable to represent the gut microbiota accurately due to their low coverage [11]. Additionally, they were time-consuming, costly, and insufficient in reflecting the true diversity of the gut microbiota [11].

Sequence-based metagenomics, involving the extraction, fragmentation, size-separation, and random direct sequencing of DNA from an environmental sample, has become the method of choice for studying microbial communities due to its high accuracy [1]. Previously, the more commonly used sequencing technique involved amplification and sequencing of either the 16S rRNA gene (for bacteria and archaea) or the internal transcribed spacer (ITS) region (for fungi) in the sample DNA [1]. However, direct shotgun sequencing of the DNA sample of the entire microbial community has become more popular due to its high sensitivity, reproducibility, and coverage. Metagenomic analysis is thus a powerful tool for studying microbial communities and their importance in various environments, including the gastrointestinal tracts of animals. This approach allows for the identification of both cultivable and noncultivable microorganisms and their associated genes, thus providing a more comprehensive picture of the microbial ecology of poultry [1].

Unfortunately, only a few studies have applied metagenomics to investigate bacterial communities and antimicrobial resistance genes (ARGs) present in poultry raised in free-range environments in Kenya. For instance, a study by Nduku et al. [12] found a high prevalence of extended-spectrum beta-lactamase (ESBL)-producing *Escherichia coli* in poultry in Kenya. However, several studies have investigated the prevalence of bacterial pathogens and ARGs in poultry elsewhere. For instance, Havelaar et al. [13] estimated that the global burden of food-borne illness due to nontyphoidal *Salmonella* in poultry was over 60 million cases yearly. In addition, a study in Poland comparing the AMR gene profiles of farm animals exposed to antimicrobial treatment to those of wild animals that seemed not to be subjected to antimicrobial pressure revealed higher levels of AMR in farm animals than in wildlife [8]. Furthermore, Skarżyńska et al. [8] underscored the potential of wildlife in disseminating AMR. In another study in China, microbial community and resistome profiles in cecal, cloacal, and fecal samples of broilers were compared to determine the feasibility and comparative merits and demerits of using particular sample types to study gut microbiota [14]. The authors observed that fecal microbiota have limited potential as a proxy in chicken gut microbial community studies. Feces should therefore be used with caution when characterizing gut microbiomes.

Most metagenomic studies on poultry microbiomes have been carried out on poultry raised under controlled and regulated feeding regimes. However, metagenomic studies on free-ranging poultry are more informative than those on poultry raised under controlled conditions [15]. This is because free-ranging poultry are exposed to a broader range of environmental conditions, which can influence their microbial communities [16]. In contrast, poultry raised under controlled conditions are exposed to a more homogeneous environment, which may limit their bacterial communities' diversity and affect their ARG profiles [17]. To our knowledge, only one study in Ethiopia investigated the microbial community profiles of indigenous backyard chickens on a scavenging feeding system from two geographically and climatically distinct regions [18]. Metagenomics analysis of poultry raised under a free-range feeding system is therefore required to explore the impact of local feed (plants, insects, and other small animals) on poultry health [18]. In addition, this aids in understanding the microbiome compositional structure of the environment in free-ranging poultry. In this study, we characterized bacterial pathogens and ARGs present in the cloacal and oropharyngeal regions of free-range poultry in Kenya using a metagenomic approach. The cloacal and oropharyngeal swab samples have been widely used for the detection of poultry pathogens because most bacterial and viral infections in birds are mainly through the fecal-oral route, making these regions a critical study area [19, 20]. Our findings will contribute to a better understanding of the gut bacterial pathogens of poultry raised in free-range environments and inform us of the interventions needed to reduce the risk of food-borne illnesses and antimicrobial resistance.

## 2. Materials and Methods

### 2.1. Sample Collection

**Figure 1:**
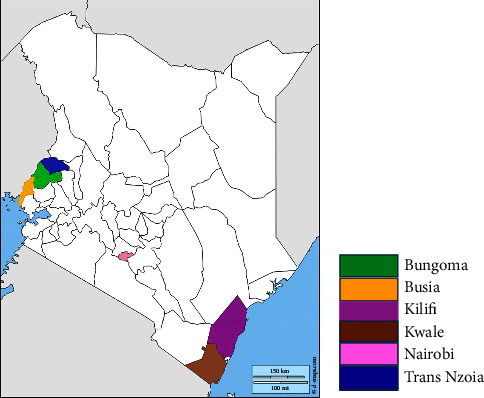
Map of Kenya showing the main sampling sites with varying geographic and climatic conditions for cloacal and oropharyngeal swab samples (*source: GeoCurrents map*).

This study was carried out from 2016 to December 2018 across six counties with varying agroecological conditions in Kenya (Figure 1). The study received institutional clearance from the Jomo Kenyatta University of Agriculture and Technology (JKUAT) to conduct animal research. Clearance was also sought from the Director of Veterinary Services from the State Department of Livestock, Ministry of Agriculture, Livestock Development, and Co-operatives, Kenya, to study farm animals. A stratified cross-sectional purposive approach was used during sample collection. The study areas were divided into subcounty populations to reduce sample bias. The maximum possible number of households per subcounty population was then considered. Households were selected based on their willingness to participate in the study. A distance of 0.5 km between households was maintained to avoid chances of sampling related individuals.

The study collected cloacal (*n* = 563) and oropharyngeal (*n* = 394) swabs from selected regions in Kenya with distinct geographic and climatic conditions. The targeted regions included counties bordering Uganda (Bungoma, Busia, and Trans Nzoia), maritime borders (Kilifi and Kwale), and urban areas of Nairobi (Figure 1). In addition, information on flock condition or performance was also collected. The collected cloacal and oropharyngeal swab samples were immediately frozen in dry ice and later placed in liquid nitrogen in the field. They were then processed in preparation for downstream analysis or permanently preserved at −80°C until processing.

### 2.2. Extraction of Nucleic Acids and Sequencing

The cloacal and oropharyngeal swab samples were processed in pools (16 pools representing the 563 cloacal swabs and 14 pools representing the 394 oropharyngeal swabs) (Supplementary Materials Tables S1 and S2).

#### 2.2.1. DNA Extraction

DNA was extracted from the pooled cloacal and oropharyngeal swabs using the PureLink Genomic DNA Mini Kit (Invitrogen, Thermo Fisher Scientific, Waltham, Massachusetts, USA) following the manufacturer's protocol. Briefly, the swab was placed into a 2 ml Eppendorf tube to which 200 *μ*l of phosphate-buffered saline (PBS) and 20 *μ*l of proteinase K were added and mixed well by pipetting. An equal volume (200 *μ*l) of PureLink^R^ Genomic Lysis/Binding Buffer was added to the lysate and mixed well by vortexing briefly before incubating at 55°C for at least 10 minutes. The lysate was briefly centrifuged at 3, 000 × g and 200 *μ*l of 99% ethanol was added and mixed well by vortexing for 5 seconds. The lysate was then added to a PureLink^R^ Spin Column attached to a collection tube and centrifuged at 10, 000 × g for 1 minute at room temperature. The collection tube was discarded, and the spin column was placed into a clean PureLink^R^ collection tube. To wash the extracted DNA, 500 *μ*l of the wash buffer 1 prepared with ethanol was added to the column and centrifuged at room temperature at 10, 000 × g for 1 minute. The collection tube was discarded, and the spin column was placed into a clean PureLink^R^ collection tube. A second washing was done by adding 500 *μ*l of wash buffer 2 to the column and centrifuged at maximum speed for 3 minutes at room temperature, and the collection tube was discarded. The spin column was finally placed in a sterile 1.5 ml microcentrifuge tube, and 50 *μ*l of PureLink^R^ Genomic Elution Buffer was added to the column, which was incubated at room temperature for 1 minute and centrifuged at maximum speed for 1 minute at room temperature. To recover more DNA, a second elution step using the same elution buffer volume as the first was performed in another sterile, 1.5 ml microcentrifuge tube. The column was then removed and discarded. The purified DNA solution was stored at a −20°C freezer until it was processed at the International Livestock Research Institute (ILRI) genomic platform where library preparation and whole genome shotgun sequencing were done.

#### 2.2.2. Sequencing

The quality and quantity of the DNA preparations were determined in the NanoDrop™ 2000 spectrophotometer and Qubit fluorometer (Invitrogen, Thermo Fisher Scientific, Inc., Waltham, Massachusetts, USA), respectively. The extracted genomic DNA was used to prepare indexed paired-end libraries using Nextera™ XT DNA Library Preparation Kit according to the manufacturer's instructions (Illumina, Inc., USA). Indexed samples were pooled and reconstituted to 4 nM before diluting to 12 pM for loading into the MiSeq instrument (Illumina, CA, USA) version 2 reagent kit (300 cycles) with a paired-end format (2 × 150 cycles) at the ILRI Genomic platform, Nairobi, Kenya. The number of reads obtained from each library is shown in Tables 1 and 2.

### 2.3. Taxonomic Assignment

The metagenomic analysis was done using the Metaphlan version 3.0 [21] and SqueezeMeta version 1.5.1 workflows [22]. Poor-quality sequencing reads (short contigs <200 bp) and adaptors were trimmed using Prinseq version 0.39 [23]. Read mapping against host references was performed to remove host DNA using Bowtie2 version 2.4.5 [24]. The paired-end sequence reads were *de novo* assembled into contigs using Megahit version 1.0.2 [25]. The assembled contigs were used for taxonomic assignment and functional annotation analyses. Taxonomical abundance was determined by comparing metagenomic reads to a database of taxonomically informative gene families to annotate each metagenomic homolog. Merged abundance tables used for the assignment of different taxonomic units were generated using Metaphlan version 3.0 [21]. Sequences were therefore classified using the RDP classifier into operational taxonomic units [26, 27]. An operational taxonomic unit (OTU)-based method was used for analysis where sequences were split into bins based on taxonomy [28–30].

The merged abundance tables were used to assign taxonomies at different levels. We then used the SqueezeMeta version 1.5.1 workflow to generate the contigs that were used to create the phyloseq object using the phyloseq package in R version 4.3.0 [31] and, consequently, the OTU table that was used for downstream analyses using the same workflow. The analysis includes plotting rarefaction curves, alpha diversity indices (for analyzing microbial community diversity and richness), and beta diversity indices (for comparison of microbial diversity between different poultry species and sample types). PCoA analysis was also performed for taxonomic assignment to determine the distances between levels of classification. Phyloseq v1.44.0 and ggplot2 v3.4.2 packages in R were used to visualize the abundance of bacterial taxonomic composition.

### 2.4. Functional Annotation

The function of the coding sequence was inferred based on similarity to sequences in the Kyoto Encyclopedia of Genes and Genomes (KEGG) as proposed by Kanehisa and Goto [32] and Clusters of Orthologous Genes (COG) databases using diamond implementation of the basic alignment search tool (BLAST) [33] with a cutoff of above 40% of the reference and query ratio being used. Clustering, principal component analysis (PCA), and nonmetric multidimensional scaling (NMDS) analyses were performed using the generated taxonomic and functional abundance tables.

### 2.5. Characterization of Antimicrobial Resistance Genes (ARGs)

Antimicrobial resistance genes (ARGs) from the poultry cloacal and oropharyngeal swab content were characterized to explore the relationship between diverse sequences and resistance levels. The assembled contigs of cloacal and oropharyngeal swabs of the different poultry species were aligned against the NCBI AMRFinderPlus [34] and Resfinder [35] databases for mass screening of the assembled contigs for ARGs using ABRicate software version 1.0.1 [36].

Based on raw read counts, the relative abundances of AMR genes were estimated. Analysis and visualization of results on graphs and heat maps were carried out in the open source RStudio 3.5.3 version for Windows (https://www.rproject.org/) using the library(vegan), library(ggplot2), library(reshape2), and library(RColorBrewer) packages. The ARGs' relative abundance between the cloacal and oropharyngeal swabs and their distribution through hierarchical clustering in all classification levels are reported.

## 3. Results

### 3.1. General Overview of the Sequence Data

A total of 17,002,195 paired-end reads (from cloacal swab samples) and 11,050,372 paired-end reads (from oral-pharyngeal swab samples), with a median length of 200 base pairs (bp), were obtained from all samples (Tables 1 and 2). The total number of clean reads generated from cloacal and oropharyngeal samples was 16,432,416 and 10,879,784, respectively. These were subsequently assembled into a total of 66,090 and 60,098 contigs, respectively. Using a 95% similarity cut-off, the assembled contigs yielded 301 and 275 operational taxonomic units (OTUs) for cloacal and oropharyngeal swabs, respectively. Three samples (CN3, CN10, and DK3) were not informative as they did not generate any OTUs that could be used for taxonomic assignment.

**Figure 2:**
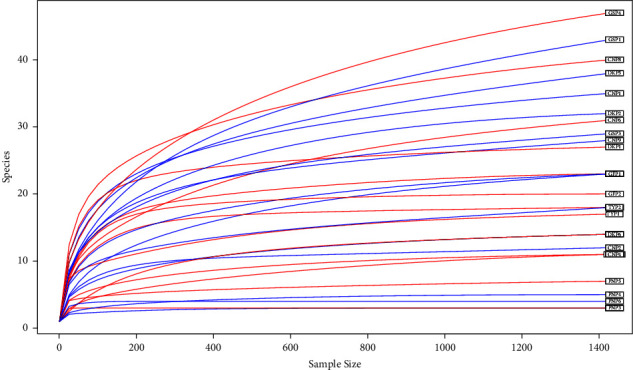
Rarefaction curves of samples clustered at 90% sequence identity. The rarefaction curves for each sample were plotted without replacement. Rarefaction is used to simulate an even number of reads per sample. In this study, the rarefaction depth chosen is 90% of the minimum sample depth in the dataset. For each poultry species and sample type, CN = chicken, DK = duck, GF = guinea fowl, GS = goose, PN = pigeon, and TY = turkey.

Rarefaction (discovery) curves generated from the OTUs show that all the samples approached a plateau, which suggests that the sample volumes were efficient in estimating both cloacal and oropharyngeal taxa (Figure 2).

Analysis of species richness (observed number of OTUs and ACE) and community diversity (Chao1, Shannon, and Inverse Simpson indices) showed that there was no significant difference in species richness and diversity in cloacal and oropharyngeal samples across the poultry species except for the pigeons, which had much lower richness and diversity compared to other species (Tables 3 and 4). This implies that the species richness and diversity of the bacterial pathogens that colonize both the cloacal and oropharyngeal regions are generally similar across the different poultry species.

**Figure 3:**
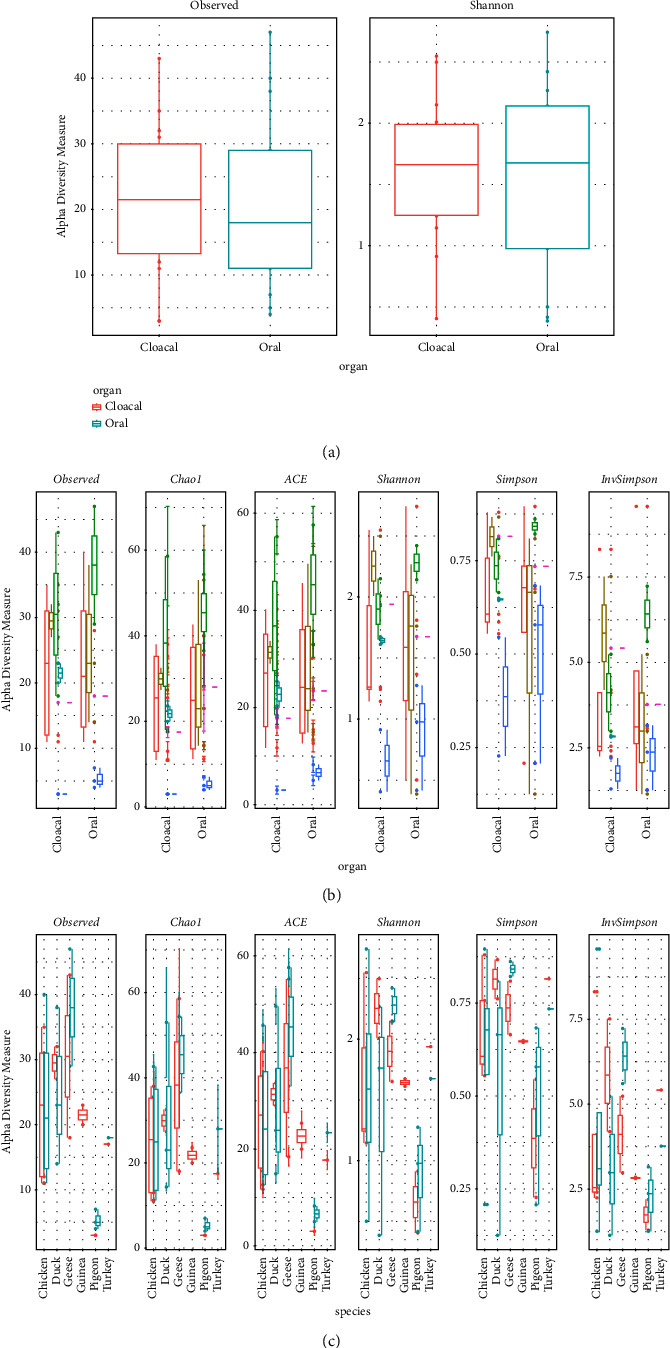
Alpha diversity measures (a) the number of OTUs and the Shannon entropy grouping of the different species by sample type; (b) alpha diversity measures by sample type; and (c) alpha diversity measures by species.

The number of OTUs and Shannon entropy groupings of the different species by sample type and other alpha diversity measures by sample type and species are shown in Figure 3. The results similarly showed that there was no marked difference in the species richness of the detected bacterial pathogens between the cloacal and oropharyngeal samples in the different poultry species.

A Wilcoxon rank-sum (Mann–Whitney) nonparametric test was used to determine whether the observed number of OTUs differed significantly between sample types. The pairwise comparisons using the Wilcoxon rank sum test with continuity correction are provided (Supplementary Materials Tables S3–S5). The results show no statistically significant differences in the diversity of microbial communities between the cloacal and oropharyngeal samples for any of the diversity indices examined.

Diversity indices were also tested to determine whether they differed significantly between species (Supplementary Materials Tables S6–S8). The results show the pairwise comparisons of species richness (observed, Shannon, and Chao1) between poultry species (chicken, duck, goose, guinea fowl, pigeon, and turkey). Based on observed richness, no significant difference was observed in the bacterial pathogen community richness between most of the poultry species (*p* > 0.05). However, in Shannon's diversity index, there is a significant difference in the bacterial species richness of pigeons compared to other species—a value of *p* < 0.05 was obtained for pigeons compared to ducks and geese. Using the Chao1 diversity index, a significant difference was observed in the richness of pigeons compared to chickens, ducks, and geese (*p* < 0.05). These results therefore suggest that pigeons have a different bacterial species richness when compared to other poultry species which do not differ significantly in species richness.

### 3.2. Cloacal and Oropharyngeal Bacterial Pathogen Composition across Poultry Species

**Figure 4:**
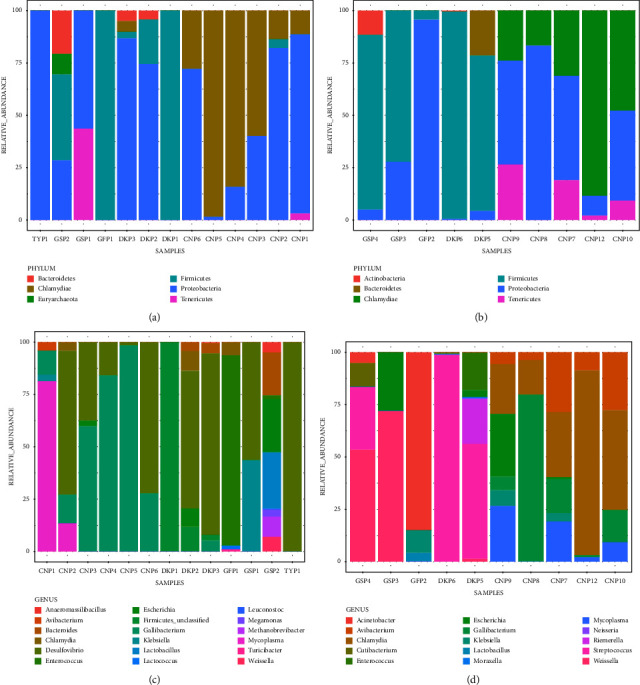
Bacterial composition at phylum and genus levels; (a) cloacal relative abundance at phylum level; (b) oropharyngeal relative abundance at phylum level; (c) cloacal relative abundance at the genus level; and (d) oropharyngeal relative abundance at genus level for the different poultry species in all samples. A stacked column chart with taxonomic relative abundances (*y*-axis) by sample (*x*-axis). The height of each bar chart relates to the taxonomic relative abundances in a sample. For each poultry species and sample type, CN = chicken, DK = duck, GF = guinea fowl, GS = goose, PN = pigeon, and TY = turkey.

Phylum and genus-level distributions for individual samples are shown in Figure 4. At the phylum level, *Proteobacteria*, *Chlamydiae*, and *Firmicutes* were the most dominant phyla detected in cloacal and oropharyngeal samples across the poultry species. *Chlamydiae* were mostly detected in chicken samples, except for one pooled sample in ducks. *Proteobacteria*, on the other hand, was detected in chickens, ducks, and geese, while *Firmicutes* was detected in ducks and geese. Other phyla that were detected in cloacal samples included *Bacteroidetes* (in ducks and geese) and *Tenericutes* (in geese and chickens). Other phyla detected in oropharyngeal samples included *Tenericutes* (in chickens), *Bacteroidetes* (in ducks), and *Actinobacteria* (in geese).

*Desulfovibrio*, *Gallibacterium*, and *Mycoplasma* were the most dominant genera across the poultry species in the cloacal swabs. Other genera detected in some poultry species included *Escherichia*, *Klebsiella*, *Chlamydia*, *Bacteroides*, *Enterococcus*, and *Avibacterium*. Most of these bacteria are potentially pathogenic. In the oropharyngeal samples, the most dominant genera were *Chlamydia*, *Escherichia*, *Avibacterium*, *Gallibacterium*, *Mycoplasma*, *Klebsiella*, and *Neisseria*, which are common etiological agents of poultry diseases.

**Figure 5:**
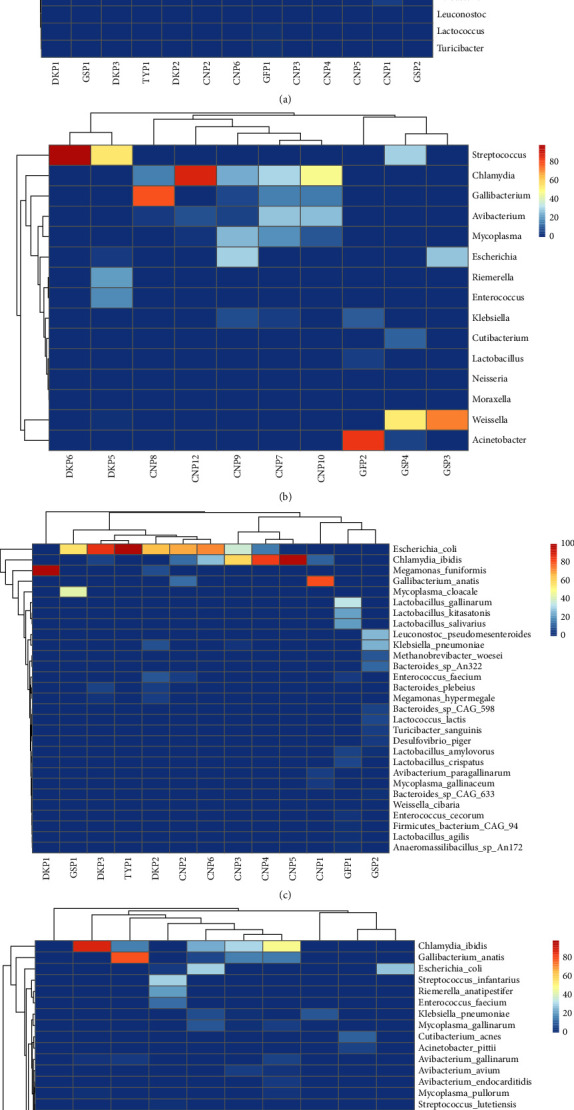
Taxonomic abundances heat map based on log-transformed relative abundance values; (a) heatmap representation of cloacal taxonomy abundance of the dominant genera; (b) heatmap representation of oropharyngeal taxonomy abundance of the dominant genera; (c) heatmap representation of cloacal taxonomy abundance of the detected species; and (d) heatmap representation of oropharyngeal taxonomy abundance of the detected species (*y*-axis) in all samples (*x*-axis). Color scale from red (high abundance) to blue (low abundance) represents log-transformed relative abundance. For each poultry species and sample type, CN = chicken, DK = duck, GF = guinea fowl, GS = goose, PN = pigeon, and TY = turkey.

To assess the relatedness and overall taxonomic similarities between the identified sequences in the cloacal and oral-pharyngeal swab samples, a hierarchical clustering analysis of the dominant genera and species of all samples for both groups was performed (Figure 5). The hierarchical cluster maps for both groups generally had dendrograms with intermingled branches, implying a lack of clear separation between samples from the different poultry species. The results therefore indicate the absence of bacterial pathogen-host specificity for most of the samples studied. However, certain bacteria were only detected in the cloaca and not the oropharynx, and vice versa. The hierarchical cluster maps also showed the dominance of *Desulfovibrio*, *Gallibacterium*, and *Mycoplasma* in the poultry cloacal swab samples. Additionally, the cluster map also showed that *Chlamydia*, *Gallibacterium*, *Avibacterium*, and *Mycoplasma* were the most dominant genera in the oropharyngeal swab samples across the poultry species. Species abundance was also resolved, revealing that *Escherichia coli* and *Chlamydia ibidis* were the most dominant bacterial species across the poultry species in cloacal samples, while *Streptococcus suis*, *Chlamydia ibidis*, *Gallibacterium anatis*, *Avibacterium paragallinarum*, *Mycoplasma gallinaceum*, and *Weissella confusa* dominated the poultry oropharynx.

**Figure 6:**
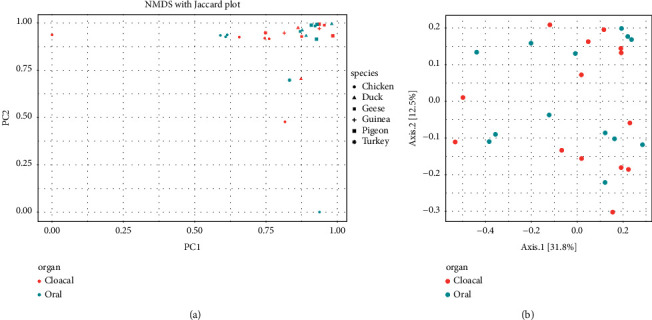
Comparison of poultry bacterial pathogens in cloacal and oropharyngeal swabs; (a) NMDS plot with Jaccard distance and (b) PCoA plot based on unweighted UniFrac distance matrices.

Beta diversity analysis was performed to investigate the diversity between sample types (cloacal and oropharyngeal samples) and also between poultry species (chickens, ducks, guinea fowls, geese, pigeons, and turkeys). The NMDS with Jaccard distance were used for dimension reduction analysis (Figure 6(a)). The NMDS plots show that there is no difference between the two groups. The principal coordinate analysis (PCoA) plot comparing the poultry microbiomes of cloacal and oropharyngeal samples by keeping parameter standard error ellipses at a 95% confidence level also revealed no clear demarcation between the microbiomes of the different poultry species or sample types (Figure 6(b)).

**Figure 7:**
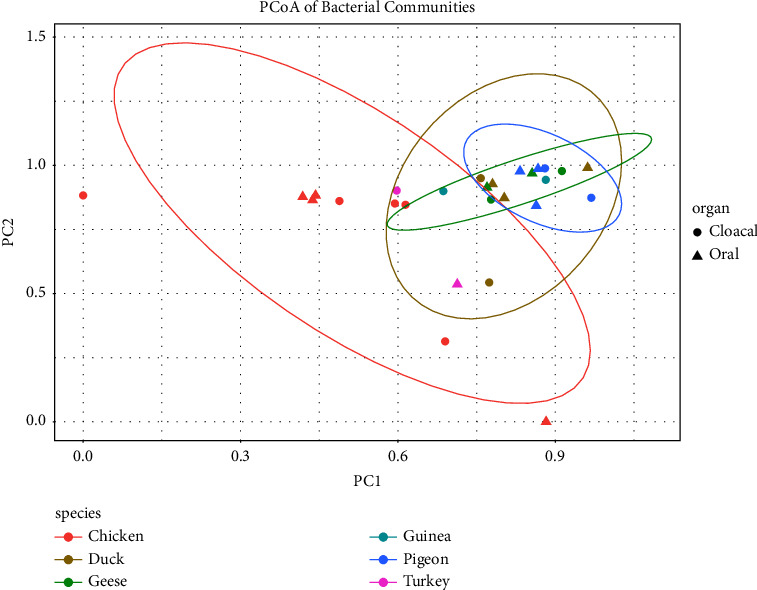
Comparison of poultry bacterial pathogens across poultry species using the PCoA plot based on the ordination of the distance matrix generated using Bray–Curtis distance.

Based on the ordination of the distance matrix generated using the Bray–Curtis complementary algorithm, a clear demarcation between bacterial assemblages from the cloaca and oropharynx was equally not apparent along the principal coordinate axis 1 (PC1) of the PCoA plot as the microbiota communities of the cloaca and oropharynx overlapped, indicating that the community structures of the two segments were similar across the poultry species (Figure 7).

The separation was confirmed using the permutational analysis of variance (PERMANOVA), which tests whether the sample types differ significantly (Table 5). The ANOVA test also suggests that the difference in diversity between the two groups is not statistically significant (*p* > 0.05).

The PERMANOVA analysis also tested whether the poultry species differ significantly from each other (Table 6). The results showed that the difference between metagenomes across the poultry species is significant (*p* < 0.05) with approximately 38% of the variations being determined by the poultry species type. The ANOVA analysis also shows that the residual variation is relatively low, indicating that the variation that is not based on the species is small.

### 3.3. Functional Annotation

**Figure 8:**
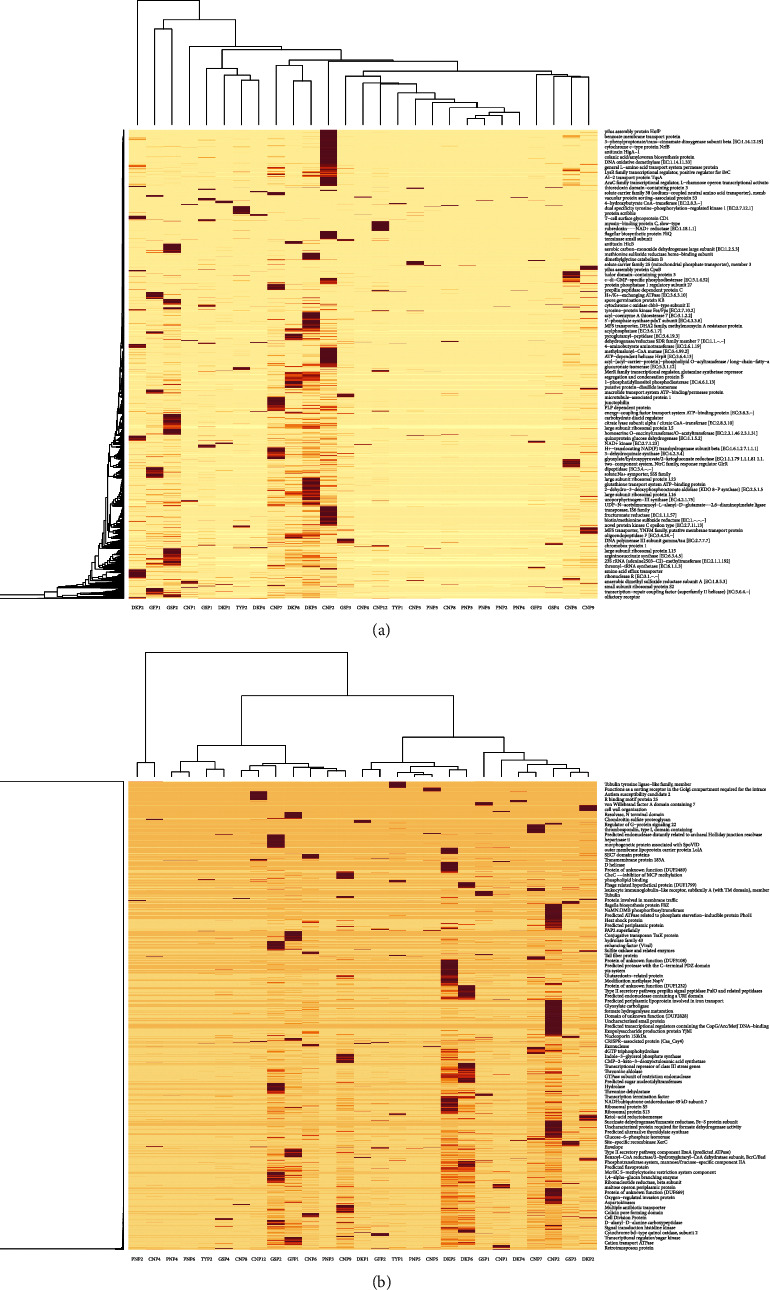
Heatmap based on log-transformed relative abundance values showing the different abundances of predicted functions; (a) sample-wise KEGG pathway distribution plot and (b) COG pathway at different taxonomic levels between the two types of microbiomes across poultry species. Color scale from red (high abundance) to white (low abundance) represents log-transformed relative abundance. For each poultry species and sample type, CN = chicken, DK = duck, GF = guinea fowl, GS = goose, PN = pigeon, and TY = turkey.

The functional diversity of a microbial community can be quantified by annotating metagenomic sequences with functions [15]. Classification of assembled metagenomic protein sequences into a protein family (function) requires searching protein family databases. We mapped protein-coding sequences against the Kyoto Encyclopedia of Genes and Genomes (KEGG) and Cluster of Orthologous Genes (COG) databases. Relative abundance in level 1 hits of each database was plotted as a heatmap of functional abundance for each sample (Figures 8(a) and 8(b)).

The KEGG pathway analysis showed that genetic information processing, environmental information processing, and cellular processes were abundant in both cloacal and oropharyngeal samples. The COG pathway analysis, on the other hand, showed that human diseases and metabolism were abundant in both cloacal and oropharyngeal samples, with functions such as cellular processes, signal transduction, environmental information processing, and information storage and processing being detected only in certain poultry species and sample types.

### 3.4. Annotation of Antimicrobial Resistance Genes (ARGs)

**Figure 9:**
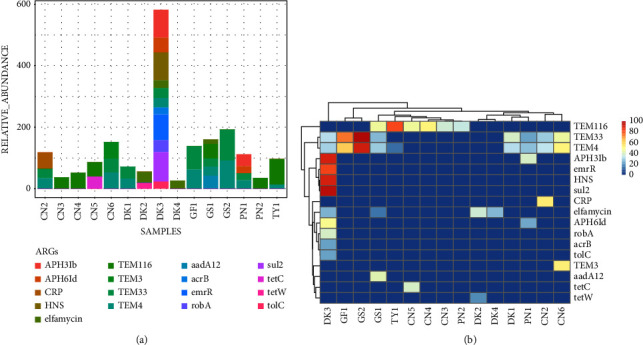
Total level of antimicrobial resistance genes in cloacal samples; (a) a stacked column chart with relative abundances of AMR genes aggregated to corresponding ARGs (*y*-axis) by sample (*x*-axis) with the height of each bar chart relating to the relative AMR gene abundances in a sample; and (b) AMR genes abundances heat map based on log-transformed relative abundance values. For heatmaps, the color scale from red (high abundance) to blue (low abundance) represents log-transformed relative abundance, and blue (0 on a scale) means no ARGs detected. For each poultry species and sample type, CN = chicken, DK = duck, GF = guinea fowl, GS = goose, PN = pigeon, and TY = turkey.

The Antimicrobial Resistance Genes Database (ARDB) was used to identify ARGs in the cloacal and oropharyngeal samples across all poultry species. Several genes responsible for AMR were detected in cloacal samples, such as those conferring resistance to beta-lactamases (*TEM116*, *TEM33*, *TEM4*, *TEM3*, and *aadA12*), tetracycline (*tetC* and *tetW*), aminoglycosides (*APH3Ib*), sulfonamides (*sul2*), and multidrug efflux pumps (*acrB*, *tolC*, and *emrR*) (Figure 9). Other proteins associated with AMR such as *HNS*, *CRP*, and *robA* were also identified. Ducks, guinea fowls, geese, and turkeys had the highest concentration of ARGs.

**Figure 10:**
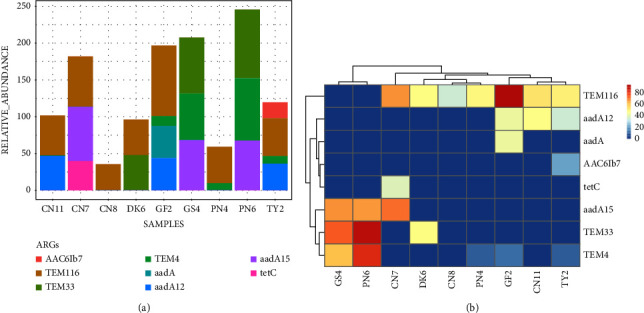
Total level of antimicrobial resistance genes in oropharyngeal samples; (a) a stacked column chart with relative abundances of AMR genes aggregated to corresponding ARGs (*y*-axis) by sample (*x*-axis) with the height of each bar chart relating to the relative AMR gene abundances in a sample; and (b) AMR genes abundances heat map based on log-transformed relative abundance values. For heatmaps, color scale from red (high abundance) to blue (low abundance) represents log-transformed relative abundance, and blue (0 on a scale) means no ARGs detected. For each poultry species and sample type, CN = chicken, DK = duck, GF = guinea fowl, GS = goose, PN = pigeon, and TY = turkey.

Just like in the cloacal samples, the major ARGs found in oropharyngeal samples also confer resistance to *β*-lactamases (*TEM16*, *TEM33*, and *TEM4*), aminoglycosides (*aadA12*, *aadA*, and *aadA15*), and tetracycline (*tetC* and *AAC6Ib7*) (Figure 10). Guinea fowls, pigeons, and geese had higher concentrations of ARGs compared to the other poultry species. Cloacal samples generally had a higher number of ARGs compared to oropharyngeal swab samples.

## 4. Discussion

Several studies have underscored the considerable impact of gut microbiomes on poultry health and performance [37, 38]. It is therefore important to evaluate the community profiles of important microorganisms colonizing the GIT of livestock, especially bacterial pathogens serving as etiological agents of livestock diseases. Metagenomic studies utilizing shotgun sequencing technologies have been used widely in recent years to study microbial populations since most microbes are not cultivable [18, 39, 40]. Additionally, the 16S rRNA gene has also been extensively used as an important phylogenetic marker for studying microbial communities [11, 39]. The main advantage that whole genome sequencing has over 16S rRNA and other marker-based sequencing methods is that it spans the entire genome of the microbes. The generated sequence can therefore be aligned against ARGs and reference genomes in different databases to identify microbes even at strain level [18] as well as genes associated with AMR. However, despite using 16S rRNA or WGS in various animals like humans, pigs, and chickens, few studies have taken advantage of metagenomic analysis to investigate bacterial pathogens affecting backyard poultry managed in unregulated or scavenging systems.

Rarefaction (discovery) curve analysis of samples shows that all the samples approached a plateau, which suggests that the sample volumes were efficient in estimating both cloacal and oropharyngeal taxa, as alluded to by Andreani et al. [41]. Pairwise comparisons of species richness (observed, ACE, Shannon, and Chao1) between different poultry species (chicken, duck, goose, guinea fowl, pigeon, and turkey) were tested to determine whether they differed significantly between species. Overall, the results suggest that pigeons have a distinctly lower species richness than other poultry species, which do not differ significantly. In a study conducted in India to investigate the molecular basis of differential host responses to avian influenza viruses in birds with differing susceptibility, it was observed that pigeons showed the lowest number of differentially expressed genes (DEGs) in most tissues, indicating a response to infection despite the low viral loads [42]. Previous studies have also shown that pigeons were highly resistant to H5N1 infections, suggesting that they have an inherent ability to prevent viruses and other pathogens from entering cells or spreading [42, 43], hence their low bacterial pathogen species richness and diversity. There were also no marked differences in species richness between cloacal and oropharyngeal samples for the different species under study. This is similar to a study by Andreani et al. [41] comparing cloacal and cecal microbiome in broiler chickens from Northern Ireland which showed that cloacal and cecal microbiomes from the same individual were more similar than expected by chance. Unfortunately, it was not possible to compare our findings on bacterial pathogen species richness and diversity to other published works on cloacal and oropharyngeal microbiomes because of insufficient literature comparing microbiomes in these two regions. We, therefore, recommend more studies to compare microbial community profiles in the cloacal and oropharyngeal regions of poultry to help understand the similarities and differences in the microbial composition and diversity in these two regions.

Our study reports that *Proteobacteria*, *Chlamydiae*, and *Firmicutes* were the most dominant phyla in the cloacal and oropharyngeal samples across the poultry species, which is consistent with the findings by Kang et al. [14] that reported *Firmicutes*, *Bacteroidetes*, and *Proteobacteria* as the dominant phyla in the poultry in the hindgut and feces, although *Bacteroidetes* were detected in lower numbers in the current study. However, our results differ from previous observations by Yan et al. [40] and Kumar et al. [18], who suggested that *Bacteroidetes* and *Firmicutes* were the most abundant phyla in chickens. They also differ from the findings by Andreani et al. [41] who found *Firmicutes* to be a proportionally more dominant phylum (∼95%) in cloacal and cecal samples of broiler chickens in Northern Ireland. However, just like Andreani et al. [41], other phyla such as *Proteobacteria*, *Tenericutes*, *Actinobacteria*, and *Bacteroidetes* were detected in lower taxa numbers. We note that the difference between our findings and those of other authors [18, 40] could be due to the differences in environment and agroclimatic conditions. It is noteworthy that while our study was on poultry raised in free-range conditions in different agroclimatic conditions, the study by Kumar et al. [18] and Yan et al. [40] investigated microbial communities in chicken under controlled conditions. Furthermore, their investigations were based on the general microbial profiles in the caeca and ilea of chicken, while the present study specifically considered the bacterial communities of pathogenic potential in the cloacal and oropharyngeal swabs of several poultry.

Our results also showed that *Desulfovibrio*, *Gallibacterium*, and *Mycoplasma* were the most dominant genera in the cloacal samples across the poultry species, with *Escherichia*, *Klebsiella*, *Chlamydia*, *Bacteroides*, and *Avibacterium* also being detected in some poultry species, albeit in lower proportions. In contrast, *Lactobacillus*, *Lachnoclostridium*, *Clostridium*, and *Bacteroides* were the dominant genera in the cecum, cloaca, and feces [14], while Enterobacteria, Lactobacilli, and Enterococci were found to dominate the small intestines of chickens in Malaysia [11]. On the other hand, *Lactobacillus* and *Bacteroides* were predominant in the small intestines of chickens in China [39]. Another study by Schreuder et al. [44] found that *Romboutsia*, *Gallibacterium*, and *Fusobacterium* were most abundant across all samples, which equally contradicted the findings of this study. Most of the bacteria detected in the current study are potentially pathogenic. In the oropharyngeal swabs, the most dominant genera were *Chlamydia*, *Escherichia*, *Avibacterium*, *Gallibacterium*, *Mycoplasma*, *Klebsiella*, and *Neisseria*, which are common etiological agents of poultry diseases.

At the species level, the hierarchical cluster maps revealed that *Escherichia coli* and *Chlamydia ibidis* were the most dominant bacterial species in the cloacal samples, while *Streptococcus suis*, *Chlamydia ibidis*, *Gallibacterium anatis*, *Avibacterium paragallinarum*, *Mycoplasma gallinaceum*, and *Weissella confusa* were detected in higher abundance in the oropharyngeal swab samples. Avian pathogenic *Escherichia coli* (APEC) causes colibacillosis, which is a severe respiratory and systemic disease in chickens [45], while *Chlamydia* infection in birds typically results in respiratory, ocular, and enteric symptoms, sometimes with a fatal outcome, although asymptomatic, latent infections are also common [46]. *Streptococcus* species are considered a part of the normal flora in poultry, with infections resulting from *Streptococcus* occurring secondary to other primary infections. These infections can be acute or subacute/chronic forms due to septicemia, although they can be successfully treated. However, it is a zoonotic agent that causes severe disease in humans and is a major pig pathogen worldwide [47]. The role of *Gallibacterium anatis* and *Avibacterium paragallinarum* as etiologic agents has also been reported [48, 49]. *Weissella confusa*, on the other hand, has been proposed as a good candidate for the development of novel direct-fed microbial products [50].

It should be noted that the comparison of OTUs and taxonomic composition between the current study and other reported studies may be affected by approaches adopted in conducting the study [11]. Other factors such as environment, treatment, feed additives, antibiotics, age, horizontal gene transfer, hygiene level, diet, poultry species, and agroclimatic considerations may also affect the poultry gut microbiome composition [11].

PCoA and NMDS plots showed no clear demarcation between bacterial communities from the cloaca and oropharynx across the poultry species under study. Our findings are consistent with observations made by Kang et al. [14] who observed that samples from the cecum clustered with those from the cloaca in microbial structure.

The KEGG and COG pathway analyses showed that cellular processes, nucleic acid metabolism, and environmental information processing were abundant in cloacal and oropharyngeal samples. These findings are corroborated by the observations made by [18], who reported that metabolism, genetic information processing, cellular processes, human diseases, and organismal systems were the dominant functions predicted at level one in the KEGG pathway analysis.

Previously, efforts to identify and characterize antimicrobial resistance involved cloning of cultured bacteria, resulting in significant losses of several potential ARGs because most bacteria are not cultivable [18]. The increasing interest in AMR research is necessitated largely by concerns about the improper use of antibiotics in many settings globally, causing uncontrolled propagation of ARGs [18]. The continued use of antibiotics in livestock and humans equally propagates the spread of ARGs, becoming a major global health issue [18]. The Antimicrobial Resistance Genes Database (ARDB) was used to identify ARGs in the cloacal and oropharyngeal samples across all poultry species. Several genes responsible for antimicrobial resistance were detected in cloacal samples, with the most predominant genes conferring resistance to beta-lactamases (*TEM116*, *TEM33*, *TEM4*, *TEM3*, and *aadA12*). Other genes detected were those conferring resistance to tetracycline (*tetC* and *tetW*), aminoglycosides (*APH3Ib*), sulfonamides (*sul2*), and multidrug efflux pumps (*acrB* and *tolC*). In addition, other proteins associated with AMR such as *HNS* and *robA* were also identified. Ducks, guinea fowls, geese, and turkeys had the highest concentration of ARGs, underscoring the importance of these poultry as disseminators of AMR. Of major concern is that a combination of these ARGs is expected to confer significantly high resistance to a wide range of antibiotics, including beta-lactams, aminoglycosides, and tetracyclines, considering that these drug classes are the mainstream antibiotics that are indicated for the prophylaxis and treatment of bacterial infections in humans and animals [51].

The major ARGs found in oropharyngeal samples confer resistance to *β*-lactamases (*TEM16*, *TEM33*, and *TEM4*), aminoglycosides (*aadA12*, *aadA*, and *aadA15*), and tetracycline (*tetC* and *AAC6Ib7*). This is contrary to the findings of a study investigating AMR in Ethiopian backyard chickens which reported that the most predominant ARGs detected were tetracycline-resistant genes like *tetQ*, *tetW*, and *tetX* [15]. Guinea fowls, pigeons, and geese had a higher concentration of ARGs in the oropharyngeal swab samples compared to the other poultry species. Cloacal samples generally had a higher number of ARGs compared to oropharyngeal swab samples, indicating that most of the microorganisms disseminating AMR in poultry species are enteric in nature. Our findings underscore the need to understand bacterial pathogens affecting poultry and also find ways to control the inappropriate use of antimicrobials since ARGs can be transmitted from poultry to humans by consuming contaminated poultry products.

As has previously been adduced by Panyako et al. [52], the study's limitation is that the data generated come from pooled samples rather than from individuals. This has the potential to reduce the epidemiological strength of the study as it affects the study's potential to evaluate specific differences within individual samples. However, this approach provides an opportunity to access the diverse metagenomes that are present in the feces and oral secretions of these populations.

## 5. Conclusion

Our study investigated poultry's cloacal and oropharyngeal bacterial pathogens from different geographical locations in Kenya. The results indicate the presence of many pathogenic bacteria in cloacal and oropharyngeal samples in the different poultry species studied, especially those belonging to the phyla *Proteobacteria*, *Chlamydiae,* and *Firmicutes*. In addition, using the KEGG and COG databases, some pathways related to metabolism, genetic information, and cellular processing were detected. We also report the abundance of ARGs that confer resistance to *β*-lactamases, aminoglycosides, and tetracycline in most of the poultry analyzed, raising concern about the dangers associated with continuous and inappropriate use of these antimicrobials in poultry production. The ARG data generated in this study provides a valuable indicator of the use of antimicrobials in poultry by smallholder backyard farmers in Kenya. In addition, it is noteworthy that although this study was conducted earlier (between 2016 and 2018), the poultry farming practices in Kenya have not changed much since then. Therefore, the information generated is still informative for managing bacterial diseases, especially in backyard poultry raised under scavenging conditions. We recommend further work that compares metagenomes of poultry raised in both free range and controlled conditions to help assess the impact of the free-range environments on microbial communities of poultry.

## Figures and Tables

**Table 1:** Number of raw reads, clean reads, assembled contigs, and observed number of OTUs identified in cloacal swab samples.

Sample	Number of raw reads	Number of clean reads	Number of assembled contigs	Number of observed OTUs
CN1	739,235	727,578	1,210	35
CN2	721,871	709,914	6,181	12
CN3	1,316,673	1,294,994	5,314	
CN4	962,476	946,762	1,312	11
CN5	1,183,395	1,164,692	3,553	23
CN6	972,083	954,980	3,704	31
DK1	1,083,848	1,064,236	2,268	27
DK2	1,866,041	1,836,634	5,244	32
DK3	1,381,344	1,064,236	11,450	
DK4	1,254,999	1,235,172	3,004	23
GF1	1,078,026	1,060,498	4,361	43
GS1	1,181,800	1,163,704	4,220	18
GS2	1,366,750	1,345,650	7,775	23
PN2	243,351	239,606	406	20
PN3	183,080	179,774	236	3
TY1	1,467,223	1,443,986	5,852	17
Total	17,002,195	16,432,416	66,090	301

**Table 2:** Number of raw reads, clean reads, assembled contigs, and observed number of OTUs identified in oropharyngeal swab samples.

Sample	Number of raw reads	Number of clean reads	Number of assembled contigs	Number of observed OTUs
CN7	1,508,846	1,486,620	9,637	14
CN8	270,122	265,624	881	40
CN9	574,055	565,378	3,586	28
CN10	2,382,576	2,348,126	13,704	
CN12	1,570,585	1,545,050	6,892	11
DK5	1,097,134	1,080,036	13,472	38
DK6	562,109	552,610	4,997	14
GF2	413,234	406,648	990	20
GS3	807,239	793,946	2,657	29
GS4	429,261	422,556	722	47
PN4	320,162	315,052	479	5
PN5	437,675	431,732	719	7
PN6	268,587	264,240	372	4
TY2	408,787	402,166	990	18
Total	11,050,372	10,879,784	60,098	275

**Table 3:** OTUs (0.05% coverage) and diversity indices from cloacal samples from different poultry species.

Sample	Number of observed OTUs	Chao1	ACE	Shannon	Inverse Simpson
CNP1	35	38.00	40.23	2.548	8.310
CNP2	12	13.00	16.09	1.261	2.544
CNP4	11	11.00	11.69	1.146	2.414
CNP5	23	25.50	27.06	1.244	2.245
CNP6	31	35.20	35.12	1.929	4.112
DKP1	27	27.33	29.03	2.498	7.513
DKP2	32	32.50	33.68	2.009	4.195
DKP4	23	23.00	23.88	1.761	2.989
GSP1	43	58.60	55.18	2.150	5.237
GSP2	18	18.00	18.42	1.651	2.985
GFP1	23	23.60	25.37	1.612	2.852
PNP2	3	3.00	3.00	0.405	1.294
PNP3	3	3.00	NaN	0.913	2.198
TYP1	17	17.50	17.75	1.939	5.419
Total	301				

**Table 4:** OTUs (0.05% coverage) and diversity indices from oropharyngeal samples from different poultry species.

Sample	Number of observed OTUs	Chao1	ACE	Shannon	Inverse Simpson
CNP7	14	14.33	15.46	1.367	3.144
CNP8	40	42.63	45.61	2.742	9.568
CNP9	28	35.50	32.88	1.810	3.072
CNP12	11	11.20	12.61	0.501	1.263
DKP5	38	53.00	49.56	2.267	5.232
DKP6	14	14.33	14.90	0.386	1.143
GFP2	20	20.00	20.00	1.674	2.985
GSP3	29	36.50	33.04	2.141	5.608
GSP4	47	54.33	57.58	2.420	7.230
PNP4	5	5.00	5.00	0.416	1.262
PNP5	7	7.00	8.243	1.276	3.157
PNP6	4	4.00	NaN	0.9769	2.372
TYP2	18	28.00	23.40	1.6760	3.767
Total	275				

**Table 5:** Comparison of differences in diversity between bacterial assemblages from the cloaca and oropharynx.

	Df	SumsOfSqs	MeanSqs	F.Model	*R* ^2^	Pr (>F)
Sample type	1	0.1156	0.11562	0.58477	0.02286	0.872
Residuals	25	4.9429	0.19772	0.97714		
Total	26	5.0585	1.00000			

**Table 6:** Permutational analysis of variance testing whether the poultry species differ significantly.

	Df	SumsOfSqs	MeanSqs	F.Model	*R* ^2^	Pr (>F)
Species	5	1.9615	0.39230	2.66	0.38776	0.001
Residuals	21	3.0970	0.14748	0.61224		
Total	26	5.0585	1.00000			

## Data Availability

The sequencing data of the cloacal and oropharyngeal swabs of the Kenyan poultry under this study have been submitted to the NCBI Sequencing Read Archive (SRA) under the bio-project PRJNA976898.

## Supplementary Materials

Supplementary Materials Table S1: pools and sampling regions for cloacal swab samples. Table S2: pools and sampling regions for oropharyngeal swab samples. Table S3: pairwise comparison of poultry metagenomes in different sample types test based on the observed number of OTUs. Table S4: pairwise comparison of poultry metagenomes in different sample types based on the Shannon diversity index. Table S5: pairwise comparison of poultry metagenomes in different sample types based on the Shannon diversity index. Table S6: pairwise comparison of species richness between different poultry species based on the observed number of OTUs. Table S7: pairwise comparison of species richness between different poultry species based on the Shannon diversity index. Table S8: pairwise comparison of species richness between different poultry species based on Chao1 diversity index.
