# Supplementary material for: Metagenomic Characterization of Poultry Cloacal and Oropharyngeal Swabs in Kenya Reveals Bacterial Pathogens and Their Antimicrobial Resistance Genes
Source: Int J Microbiol. 2024 Feb 12;2024:8054338. doi: 10.1155/2024/8054338 (PMC10876313; doi:10.1155/2024/8054338)
Supplement: Supplementary Materials — Table S1: pools and sampling regions for cloacal swab samples. Table S2: pools and sampling regions for oropharyngeal swab samples. Table S3: pairwise comparison of poultry metagenomes in different sample types test based on the observed number of OTUs. Table S4: pairwise comparison of poultry metagenomes in different sample types based on the Shannon diversity index. Table S5: pairwise comparison of poultry metagenomes in different sample types based on the Shannon diversity index. Table S6: pairwise comparison of species richness between different poultry species based on the observed number of OTUs. Table S7: pairwise comparison of species richness between different poultry species based on the Shannon diversity index. Table S8: pairwise comparison of species richness between different poultry species based on Chao1 diversity index. [file 8054338.f1.docx]

# Supplementary Materials: Metagenomic Characterization of Poultry Cloacal and Oropharyngeal Swabs in Kenya Reveals Bacterial Pathogens and their Antimicrobial Resistance Genes

| **Table S1**: Pools and sampling regions for cloacal swab samples |
| --- |
| Species Pool Number of samples Region |
| Chicken CN1 54 Kilifi  CN2 12 Kilifi  CN3 39 Kwale  CN4 77 Kwale  CN5 99 Nairobi, Trans Nzoia  CN6 45 Busia  Duck DK1 19 Kilifi  DK2 31 Kilifi  DK3 18 Kwale  DK4 32 Bungoma, Busia, Trans Nzoia  Guinea fowl GF1 30 Bungoma, Kilifi, Kwale  Goose GS1 28 Kilifi, Kwale  GS2 21 Bungoma, Busia  Pigeon PN2 28 Bungoma  PN3 16 Busia  Turkey TY1 14 Kilifi  **Total 16 563** |

| **Table S2**: Pools and sampling regions for oropharyngeal swab samples |
| --- |
| Species Pool Number of samples Region |
| Chicken CN7 9 Kilifi  CN8 25 Kilifi  CN9 28 Kwale  CN10 36 Kwale  CN12 47 Busia, Trans Nzoia  Duck DK5 52 Kilifi, Kwale  DK6 30 Bungoma, Busia, Trans Nzoia  Guinea fowl GF2 23 Bungoma, Kilifi, Kwale  Goose GS3 28 Kilifi, Kwale  GS4 21 Bungoma, Busia  Pigeon PN4 31 Kilifi  PN5 30 Bungoma  PN6 18 Busia  Turkey TY2 16 Busia, Kilifi  **Total 14 394** |

| **Table S3**: Pairwise comparison of poultry metagenomes in different sample types based on the observed number of OTUs | |
| --- | --- |
|  | cloacal |
| oral | 0.93 |

| **Table S4**: Pairwise comparison of poultry metagenomes in different sample types based on the Shannon diversity index | |
| --- | --- |
|  | cloacal |
| oral | 0.87 |

| **Table S5**: Pairwise comparison of poultry metagenomes in different sample types based on the Shannon diversity index | |
| --- | --- |
|  | cloacal |
| oral | 0.79 |

| **Table S6**: Pairwise comparison of species richness between different poultry species based on the observed number of OTUs. P value adjustment method: Holm | | | | | |
| --- | --- | --- | --- | --- | --- |
|  | Chicken | Duck | Goose | Guinea fowl | Pigeon |
| Duck | 1.000 | - | - | - | - |
| Goose | 1.000 | 1.000 | - | - | - |
| Guinea fowl | 1.000 | 1.000 | 1.000 | - | - |
| Pigeon | 0.049 | 0.167 | 0.253 | 0.944 | - |
| Turkey | 1.000 | 1.000 | 1.000 | 1.000 | 0.944 |

| **Table S7**: Pairwise comparison of species richness between different poultry species based on the Shannon diversity index. P value adjustment method: Holm | | | | | |
| --- | --- | --- | --- | --- | --- |
|  | Chicken | Duck | Goose | Guinea fowl | Pigeon |
| Duck | 1.00 | - | - | - | - |
| Goose | 1.00 | 1.00 | - | - | - |
| Guinea fowl | 1.00 | 1.00 | 1.00 | - | - |
| Pigeon | 0.41 | 1.00 | 0.24 | 1.00 - | - |
| Turkey | 1.00 | 1.00 | 1.00 | 1.00 | 1.00 |

| **Table S8**: Pairwise comparison of species richness between different poultry species based on Chao1 diversity index. P value adjustment method: Holm | | | | | |
| --- | --- | --- | --- | --- | --- |
|  | Chicken | Duck | Goose | Guinea fowl | Pigeon |
| Duck | 1.00 | - | - | - | - |
| Goose | 1.00 | 1.00 | - | - | - |
| Guinea fowl | 1.00 | 1.00 | 1.00 | - | - |
| Pigeon | 0.05 | 0.17 | 0.25 | 0.94 - | - |
| Turkey | 1.00 | 1.00 | 1.00 | 1.00 | 0.94 |
